# Evaluation of steroid hormones and their receptors in development and progression of renal cell carcinoma

**DOI:** 10.15586/jkcvhl.2014.9

**Published:** 2014-06-15

**Authors:** Nigel C Bennett, Retnagowri Rajandram, Keng Lim Ng, Glenda C Gobe

**Affiliations:** 1 Centre for Kidney Disease Research, University of Queensland School of Medicine, Translational Research Institute, Woolloongabba, Brisbane, Australia; 2 Oral Cancer Research Program, UQCCR, Royal Brisbane and Women’s Hospital, Herston, Brisbane, Australia; 3 Dept Surgery, Faculty of Medicine, University Malaya, Kuala Lumpur, Malaysia; 4 University Malaya Cancer Research Institute (UMCRI), Kuala Lumpur, Malaysia

## Abstract

Steroid hormones and their receptors have important roles in normal kidney biology, and alterations in their expression and function help explain the differences in development of kidney diseases, such as nephrotic syndrome and chronic kidney disease. The distinct gender difference in incidence of renal cell carcinoma (RCC), with males having almost twice the incidence as females globally, also suggests a role for sex hormones or their receptors in RCC development and progression. There was a peak in interest in evaluating the roles of androgen and estrogen receptors in RCC pathogenesis in the late 20^th^ century, with some positive outcomes for RCC therapy that targeted estrogen receptors, especially for metastatic disease. Since that time, however, there have been few studies that look at use of steroid hormone modulators for RCC, especially in the light of new therapies such as the tyrosine kinase inhibitors and new immune therapies, which are having some success for treatment of metastatic RCC. This review summarises past and current literature and attempts to stimulate renewed interest in research into the steroid hormones and their receptors, which might be used to effect, for example, in combination with the other newer targeted therapies for RCC.

## Introduction

**Figure 1.**
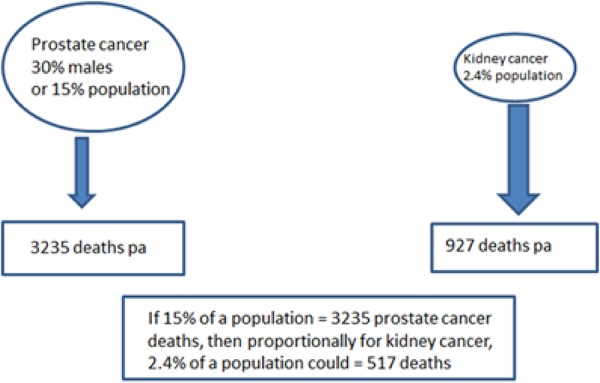
**Comparison of incidence and deaths in prostate versus kidney cancer per annum in Australia in 2010.** In 2010 there were 3,235 deaths from prostate cancer and 927 deaths from kidney cancer in Australia (1). The comparable incidence of the cancers is 30% of all males having prostate cancer diagnoses, versus kidney cancer at approximately 2.4% of a population. This indicates that kidney cancer deaths are almost double what could be expected by simply extrapolating from cancer incidence and prostate cancer deaths.

The incidence of kidney cancer is steadily increasing, and this is not only related to its increased diagnosis from abdominal imaging which is used frequently in today’s diagnostic medicine, but also to a real increase in kidney cancer incidence. Kidney cancers are the second most-commonly diagnosed urologic malignancy in Australia behind prostate cancer. Ninety-two percent of kidney cancers are renal cell carcinoma (RCC). Contrasting with prostate cancer, in 2010 there were 3,235 deaths from prostate cancer and 927 deaths from kidney cancer in Australia (1). Thus the annual deaths from prostate cancer are approximately 3.5 times kidney cancer deaths. The comparable incidence of the cancers, however, at 30% of all males having prostate cancer diagnoses, versus kidney cancer at approximately 2.4% of a population, indicates the deaths from each cancer is disproportional (Figure 1) with kidney cancer deaths almost double what could be expected extrapolating simply from prostate cancer incidence and deaths. Continuing this comparison, the 5-year overall survival of patients with prostate cancer is 92% whereas for kidney cancer is 71.9%. RCC are also highly metastatic, with approximately 25–30% of RCC patients having metastases at presentation, and 40% to 50% developing metastases after the primary treatment. The 5-year survival of patients with metastatic disease is less than 10%. However, despite its lethality compared with incidence, and poor long-term survival, kidney cancer has not yet attracted the same level of public health attention and research funding as other cancers, such as prostate cancer. The outlook for patients with RCC, especially metastatic RCC, continues to be poor.

RCC consists of three major subtypes (clear cell RCC at 70–80% incidence; papillary RCC at 10–15% incidence; chromophobe RCC at 5% incidence) (2). RCC lethality undoubtedly develops because of late detection of the cancer and clever kidney compensatory mechanisms. As shown in the tumour (T) staging of kidney tumours in the tumour-node-metastasis (TNM) system (Table 1), stage T1 kidney tumours, considered (apart from T0) least dangerous in tumour staging of renal growths, can be up to 7cm across (T1a up to 4cm, T1b from 4 to 7cm) (3) within this staging. These relatively large masses are often detected incidentally as part of abdominal imaging. Why would a 7cm mass not be detected via loss of renal function or development of pain? The kidneys have a great propensity to compensate for, and mask, focal loss of renal function. General loin pain, typical of RCC presence, is non-informative as to the source. In addition, loin pain, loin mass and frank haematuria present in less than 30% RCC patients. The late RCC detection then produces some intrinsic problems. The greater the renal mass, the more likely that the cancer may invade locally or metastasise to another part of the body. Surgical resection of the primary tumour is broadly touted as successful as a curative treatment, but is the frequent metastases that are dangerous, with approximately 50% of patients eventually developing metastatic RCC (4). Although there is some progress, there are currently no clinically-proven adjuvant therapies that exist for RCC patients at high risk of relapse following surgical resection, and treatment of the metastases is generally unsuccessful in the long term. This cancer also exhibits resistance to chemotherapy and radiation, and less than 10% of patients suffering from metastatic disease survive 5 years after diagnosis.

**Table 1.** Tumour (T) stages of kidney cancer in the TNM system of classification

T0	No evidence of a primary tumour in the kidney
T1	Tumour is no more than 7cm across and is completely inside the kidney (T1a indicates the tumour is equal to or less than 4cm across; T1b indicates a tumour of 4 to 7cm across)
T2	The tumour is more than 7cm across, but is still completely inside the kidney
T3	The cancer has spread through the outer covering of the kidney (the capsule), to a major vein, the adrenal gland or other tissues around the kidney
T4	The cancer has spread further than the tissues around the kidney

Some clinical observations such as the significant sex differences of RCC (twice as common in men as in women) (5,6), its variation with the cessation of gonadal activity, and the regression of metastatic kidney cancer during administration of progestin or androgen (greater in men than in women) has led to the hypothesis that some human kidney cancers are hormone-dependent (7). The exact nature of the role of hormones and their receptors in RCC development has yet to be clearly defined. A better understanding of the molecular pathways that determine development and progression of RCC is needed. Further investigations into steroid hormone pathobiology in RCC will greatly enhance our understanding of why kidney cancers grow and thrive, and just as importantly, resist therapies. This mini review updates information on the role of steroid hormones in kidney cancer and their possible modulation as a treatment, especially for metastatic disease.

## Steroid hormones and RCC

Steroid hormones and their receptors have an important role in normal kidney biology. The mineralocorticoid aldosterone, of the renin-angiotensin-aldosterone system (RAAS), is probably best known of the steroid hormones in kidney biology (8). The RAAS is necessary for early and late stages of kidney development. Aldosterone is endogenously secreted to maintain the RAAS. This hormone is, however, pathogenic when dysregulated. Aldosterone acts at the mineralocorticoid receptor (9). When the steroid is dysregulated, it is best known for its contribution to the development and progression of cardiovascular and renal disease. There is little direct evidence for dysregulation of the RAAS in RCC development and any links are at best suggestive, but the links to lipid biosynthesis, cholesterol metabolism and, consequently, to aldosterone support an association with clear cell RCC (ccRCC), which form the majority of kidney cancers. ccRCC are characterized by the accumulation of cholesterol, cholesterol esters, other neutral lipids and glycogen. One clue to this relationship has come from the elucidation of the hereditary kidney cancer gene, TRC8, which functions partly to degrade key regulators of endogenous cholesterol and lipid biosynthesis, thereby contributing to the changes that are typical of ccRCC. Yakirevich et al (9) have also shown that mineralocorticoid receptors are upregulated in the distal tubular epithelium during kidney cancer development. The distal tubular epithelium is the site of origin of chromophobe RCC and perhaps oncocytoma, two of the renal neoplasms with low incidence.

In addition to these studies, other epidemiological studies have found higher cancer mortality in hypertensive patients. One established risk factor for development of RCC is hypertension (5). Mortality is particularly elevated in hypertension associated with a stimulation of the RAAS. This may relate to angiotensin II being genotoxic due to its induction of oxidative stress, but aldosterone also has oxidative effects that could contribute to DNA damage and mutations (10). In kidney cancer cell culture experiments, aldosterone concentrations starting from 10nM caused a significant increase in DNA damage and the formation of micronuclei. Addition of the steroidal mineralocorticoid receptor antagonist spironolactone reduced the DNA damage and superoxide radicals, indicating a receptor-dependent process. Thus one of the most common pathways in kidney metabolism could potentially contribute to the sporadic development of RCC. Because of its broad role in our general metabolism, it is unlikely to be successful as a target for therapy.

Other steroid receptors are glucocorticoid receptor, progesterone receptor, androgen receptor (AR), estrogen receptor (ER), vitamin D receptor, and retinoic acid or retinoid X receptors (RAR, RXR). These receptors have important roles in growth and differentiation in neoplastic and non-neoplastic states. They act as ligand-dependent transcription factors, but there is growing evidence that these receptors can also induce gene expression through ligand-independent pathways (11). All have some recorded role in normal kidney growth and RCC development (12), although often the differential expression in RCC appears to be functionally-insignificant, because modulation of the receptors produces little therapy benefit to stop growth of RCC. In this mini review, we will concentrate on AR, ER, and the retinoid receptors.

## Androgen receptor in RCC

Androgens, such as testosterone, are hormones that are important for normal male sexual development before birth, during puberty, and maintenance of the male phenotype during adulthood. Androgen binding to AR results in the formation of an AR transcription-regulatory complex, whereby AR binds directly to DNA and regulates the activity of androgen-responsive genes (13). Androgens and AR have important functions in males, such as development of male-specific phenotypes during embryogenesis, spermatogenesis, sexual behaviour, and fertility during adult life, and also in females, such as development of female reproductive organs and their functions (ovarian folliculogenesis, embryonic implantation, and uterine and breast development) (14). The effects of androgens on kidney cancer development and growth have engendered interest for decades, and may be mediated through modulation of AR. However, there is also a close link between AR activity, transcription of specific cytokines and growth factor, and stimulation of their receptors, and these may also be targets for therapy in AR modulation. A deeper, improved, knowledge of the interaction between sex hormones and progression of kidney cancer is important, as use of selective steroid receptor modulators may yet represent a therapeutic option for patients with kidney cancer.

Epidemiological studies indicate that RCC has a gender difference in tumour incidence (male-to-female ratio 1.6:1) (15). The gender difference in RCC susceptibility is one of the most consistent findings in kidney cancer epidemiology. Early studies suggest that many types of diseases with incidence of gender difference might be linked to steroid hormone receptor expression and function. Examples include hematologic malignancies, and autoimmunity diseases more commonly found in women. However, early studies on the linkage between AR expression and RCC progression have produced controversial findings (12). The early studies often did not differentiate AR expression in different RCC subtypes, and AR function was often disregarded. More recently, however, Zhu et al (7) investigated the expression of AR in patients with RCC with different clinical stages and pathologic grades. They also explored the function of AR using human RCC cells in culture. The expression of AR was detected by immunohistochemistry in 44 adjacent normal kidney tissues of nephrectomies from 120 RCC patients and also in 16 metastatic RCC patients with their respective primary and metastatic tissue samples. In commonly used human RCC cell lines and normal kidney epithelial cells, expression of AR was assessed by Western blot, as well as transcriptional activity by luciferase assay in those AR-positive RCC cells. The expression rate of AR was higher in adjacent normal kidney than in RCC tissues, and it was negatively associated with tumour stage and Fuhrman’s grade. Specifically, there were 40.7% AR-positive cases in T1 compared with 8.0% in T3 (p=0.013), and 50.0% of grade I cases were found to be AR positive compared with 12.9% in grade III (p=0.008). AR expression was slightly higher in primary RCC tissues (12.5%) than their respective metastases (0%, p=0.484). Although lacking significance, the lack of AR-positive cases in the metastatic counterparts of the tumour samples may indicate that once RCC metastasize from their primary site, their AR expression level becomes even lower. There was no significant difference of AR-positivity between male and female patients in RCC or adjacent normal kidney tissue samples. AR strongly expressed in CaKi-2 and OSRC-2 RCC cells in culture, but with little transactivation, which might indicate functionally-inactive AR in those 2 RCC cell lines. These results demonstrate that, although a very heterogeneous cancer, there is a population of RCC patients in which AR might be targeted for therapy. However, more importantly, the results also indicate the need for further examination of AR function in RCC, to make a correct correlation between AR and RCC progression.

Other studies provide disparate results. A relatively high rate of AR expression (5 in 12 primary RCC and 1 in 5 metastatic RCC) was recorded (16). In contrast, other studies found: few cases that were AR-positive (17) (compared with, for example, AR expression in prostate cancer); AR expression in 3 of 21 RCC (18); and AR expression in 13 of 41 RCC cases (19). Langner et al (20) produced a systematic study that demonstrated AR in almost 27 of 182 of RCC cases (15% of the tumours). AR expression was significantly associated with low stage, well or moderately differentiated tumours, and a favourable outcome, decreasing with tumour growth and dedifferentiation. Although this study provided some important information about AR in RCC progression, similar to Zhu et al (7), it did not show AR differences between normal kidney and RCC, between primary RCC and metastasis, and between male and female RCC patients.

## Estrogen receptor and RCC – including studies of tamoxifen therapy

Female sex is generally associated with a better clinical outcome in kidney diseases (6) and the incidence of RCC in females is much lower than in males. Female hormones, such as estrogen, may play important roles in protecting females against kidney disease, including during RCC carcinogenesis, thereby accounting for the different incidence rates between males and females. The initial experiments describing differences in ER in RCC versus normal kidney, and in male versus female RCC, were carried out in the early 1980s (21–23). More recently, Yu et al (24) investigated ER in RCC compared with breast cancer cell lines. They found that ERβ was more highly expressed in RCC cell lines (A498, RCC-1, 786-O, ACHN, and CaKi-1) than in breast cancer cell lines (MCF-7 and HBL-100); however, they detected no AR or ERα by Western blot. They also reported that estrogen-activated ERβ acted as a tumour suppressor, decreasing cell proliferation and increasing apoptosis. Proliferation of RCC cell lines was significantly decreased after estrogen (17-β-estradiol) treatment. They suggested (i) that these results may explain the different RCC incidence rates between males and females; and (ii) that ERβ might be a useful prognostic marker for RCC progression and a novel developmental direction for improvements in RCC treatment.

The links with ER in RCC were also utilized when tamoxifen therapy became available as a common treatment for ER-positive breast cancer, and was applied in RCC patients as well. The outcome in RCC patients was disparate. Most studies were carried out in the 1990s. In a study by Papac and Keohane (25), 34 patients with progressive advanced RCC were treated with high-dose tamoxifen (100 mg/m^2^ daily) until progression. The overall remission rate was 12%, including 1 complete remission. Seventeen of these patients (50%) had minor remission or no change. Taking into consideration the documented progression prior to tamoxifen, further tumour growth could be arrested in 62% of the patients, with a one-year survival rate of 41%. Patients with only pulmonary metastases, good performance status and prior nephrectomy seemed to have a better survival. Side effects were comparable to those of conventional doses of tamoxifen (20–30 mg/m^2^ daily). These data suggested that high dose tamoxifen may be a useful therapeutic approach with low toxicity, especially in metastatic RCC that has few treatment options.

Henricksson et al. (26) compared interleukin 2/interferon-alpha (IL-2/IFN-alpha) and tamoxifen, with a control arm of tamoxifen only, in RCC patients. Tamoxifen had been shown to potentiate *in vivo* anti-tumour activity of IL-2, and because of its non-toxic behaviour it was included in the therapy groups. The final survival analysis showed no significant differences between the two treatment arms in comparing patients from the day of diagnosis of primary disease, from the day of first evidence of metastatic spread, or from the onset of treatment.

In another phase II study (25), the effectiveness of hormonal therapy using combined high dose androgen and provera or tamoxifen, in patients with advanced RCC, was studied. 30 patients with metastatic RCC received testosterone propionate (100 mg intramuscularly/i.m.) 5 times weekly plus provera (400 mg, i.m.) twice weekly until disease progression developed. Twenty patients, who had previously failed to respond to androgen and provera, received tamoxifen (100 mg/m^2^ daily). Of the 30 patients treated with androgen and provera, 3 developed partial responses of brief duration; 2 of 20 patients experienced tumour response with tamoxifen, with one instance of complete disappearance of pulmonary metastases, and another case demonstrating disease stability.

Thus, while combined hormonal therapy offered little therapeutic advantage in advanced RCC, tamoxifen in high dose exerted some anti-tumour effects in a small number of cases.

## Retinoids

The processes of tissue injury and repair in renal disease do not depend on the action of single genes but involve a complex network of growth factors and cytokines. In this context, retinoids, which are steroidal derivatives of vitamin A, have attracted attention as potential therapeutic agents (27). Retinoids modulate the function of several key transcription factors which are involved in mechanisms that are operative in renal diseases including kidney cancers. The link between retinoids and steroids had not been appreciated until the retinoid receptors were discovered. Retinoic acid receptors (RAR) belong to a gene family of ligand-inducible transcriptional regulatory factors that includes steroid hormone, thyroid hormone, and vitamin D3 receptors, as well as the peroxisome proliferator activated receptors. The RAR and the retinoid X receptors (RXR) can be subdivided into isotypes -α, -β, and -γ. These subtypes are expressed in a tissue- and developmental stage-specific pattern. RARα is the most widely expressed retinoid receptor isotype. In the kidney, both RAR and RXR are cell-specifically expressed. The kidney is, therefore, potentially responsive to activation of both receptors by their respective agonists. Double knock-out mouse models (both RAR and RXR deleted) have several renal malformations that include agenesis, hypoplasia, or aplasia of the ureteral bud. There are few studies, however, that look at these receptors as potential causes of cancer development and targets for RCC therapy.

Most abnormalities in retinoid receptors have been found in RAR-α and RAR-β (28). Point mutations in RAR-α were reported for some cancer cells in culture but it is not clear whether these occur spontaneously in cancers in patients, or as a result of resistance to treatment with retinoic acid. The mechanisms responsible for RAR-β alterations are not well-described but may occur as a pre-malignant change. Again, many of the investigations have been carried out in vitro, but DNA extracted from paraffin sections of tumours indicated loss of RAR-β in tumour samples. RAR-β expression may depend in the cellular level of retinoids as this receptor is inducible by retinoids. Thus, vitamin A deficiency may have a secondary effect of lowering expression of RAR-β. RAR-β mRNA was not found to be expressed by retinoid-resistant RCC cell lines but was present in a retinoid-sensitive renal cancer cell (SK-RC-06) and increased following incubation with retinoic acid. At least for RCC, this suggested that the antitumour action of RA was mediated through RAR-β (29). Berg et al (30) extended this study with a patient cohort. They found RAR-β transcripts increased in tumour cells of RCC patients who clinically responded to retinoid-based therapy, and suggested that retinoids that potently induce RAR-β expression should be evaluated in the treatment of advanced RCC.

In other similar early studies on the benefits, or otherwise, of retinoic acid therapies, Motzer et al. (31) applied their knowledge of changes in retinoid receptors in kidney cancers to carry out a phase II trial of IFN-2a and 13-cis-retinoic acid (CRA) in patients with RCC. They also performed in vitro studies to investigate potential mechanisms of interaction. IFN-2a was given daily at 3 million units (MU) and escalated to 6 and 9MU if tolerated. CRA was given at 1 mg/kg/d. Thirteen of 43 assessable patients achieved complete [3] and partial [10] responses at sites including bone metastases and renal primary tumours. Seven responding patients remained progression-free at 10 to 19 months post-therapy. The response proportion was higher than with IFN-2a alone, which was 10% in 149 patients. Thus, IFN-2a and CRA showed anti-tumour activity in patients with advanced RCC, and the proportion and nature of response suggested CRA added therapeutic benefit to IFN.

This same group progressed to a randomized phase III trial of IFN-2a with and without CRA (32). 284 patients were randomized to treatments. IFN-2a was given daily subcutaneously, starting at a dose of 3MU and escalated every 7 days from 3 to 9MU (by increments of 3MU), if toxicity was tolerated. Patients randomized to combination therapy were given oral 13-CRA 1 mg/kg/d plus IFN-2a. Complete or partial responses were achieved by 12% of patients treated with IFN2a plus CRA and 6% of patients treated with IFN2a (p=0.14). Median duration of response (complete and partial combined) in the group treated with the combination was 33 months (range, 9 to 50 months), versus 22 months (range, 5 to 38 months) for the second group (p=0.03). 19% of patients treated with IFN2a plus CRA were progression-free at 24 months, compared with 10% of patients treated with IFN2a alone (p=0.05). There was no difference in survival between the two treatment arms (p=0.26). Treatment, particularly the combination therapy, was unfortunately associated with decreased quality of life because of the toxicities of both IFN and CRA, and these trials have not progressed.

While the use of retinoic acid has not been taken up generally in RCC therapies, studies point to the use of the receptors, particularly RAR-β, as biomarkers for clinical response to therapy with retinoic acid. This work was originally carried out in pre-malignant oral lesions and cancers of the oral cavity (33). The expression of RAR-β mRNA was selectively lost in premalignant oral lesions and could be restored by treatment with isotretinoin. Restoration of the expression of RAR-β mRNA was associated with a positive clinical response, and the restoration may have a role in mediating the response to retinoids. Thus, RAR-β was a useful intermediate biological marker in trials of these agents for the prevention of oral carcinogenesis. Similar investigations in RCC patients may also be worthwhile.

## Summary

Kidney cancers such as RCC are not given the research priority they deserve. At 2–3% of all cancers, kidney cancer is seen as having low incidence. The 5-year survival of RCC patients with metastatic disease, however, is less than 10%. It seems likely that the promotion of research into other more common cancers such as breast, prostate, skin, and head and neck cancers has overshadowed the need for financial support for kidney cancer research, and this is now being reflected in the limited number of publications on such potentially-important aspects of RCC development and progression, as the role of steroid hormones and their receptors. Further investigations into steroid hormone pathobiology in RCC may greatly enhance our understanding of why kidney cancers grow and thrive, and just as importantly, resist therapies. RCC are highly heterogeneous, therefore expression patterns of any molecules (biomarkers), and response to therapy, need to be interpreted in terms of such heterogeneity of RCC. This review has attempted to stimulate renewed interest in research into the steroid hormones and their receptors in RCC.
